# Phylogenetic relationship and characterization of the complete mitochondrial genome of *Colias fieldii* (Lepidoptera: Papilionoidea: Pieridae)

**DOI:** 10.1080/23802359.2021.1944379

**Published:** 2021-06-28

**Authors:** Wenrong Xian, Yunxiang Liu, Yongqiang Ma, Hong Zhou, Xiang Song

**Affiliations:** a Qinghai Academy of Agriculture and Forestry, Qinghai University, Xining, China; b State Scientific Observing and Experimental Station of Crop Pest in Xining, Ministry of Agriculture, and Provincial Key Laboratory of Agricultural Integrated Pest Management in Qinghai, Xining, China; c School of Life Sciences, Gannan Normal University, Ganzhou, China

**Keywords:** Lepidoptera, Pieridae, mitochondrial genome, *Colias fieldii*, phylogenetic analysis

## Abstract

*Colias fieldii* is a common seen diurnal butterflies in the fields and widely distributed in many provinces of China. In this study, we sequenced and analyzed the complete mitochondrial genome (mitogenome) of *C. fieldii*. This mitogenome was 15,150 bp long and encoded 13 protein-coding genes (PCGs), 22 transfer RNA genes (tRNAs), and two ribosomal RNA unit genes (rRNAs). The overall base composition of the mitogenome was estimated to be A 39.8%, T 41.2%, C 11.4% and G 7.6%, with a high A + T content of 81.0%. Except for *cox1* started with CGA, all other PCGs started with the standard ATN codons (seven ATG, four ATT and one ATC). Most of the PCGs terminated with the stop codon TAA or TAG, whereas *cox1*, *cox2*, *nad5* and *nad4* end with the incomplete codon T––. Phylogenetic analysis showed that *C. fieldii* is indeed the sister species of *Colias erate* with a high support value. All seven Coliadinae species formed one clade and was sister to Pierinae butterflies. Within Coliadinae, the relationships (*Eurema* + (*Gonepteryx* + (*Catopsilia* + *Colias*))) were highly supported.

The genus *Colias* contains 84 species and almost 120 subspecies. They are adapted to different habitats and can be divided into mountain species, lowland species, and species inhabiting medium elevations in generally speaking (Stella et al. 2018). The wings of *Colias* butterflies are red, orange, yellow, or greenish, often with a black rim on the outer side. *Colias* butterflies occur throughout the Holarctic, including the arctic regions, and sexual dimorphism or polymorphism is expressed in this genus (Srygley and Kingsolver 1998). *Colias fieldii* Ménétriès, 1855 is a butterfly which has bright orange color wings, and it can damage soybean, alfalfa and trefoil. Mitogenome can be utilized in research on population genetic structure, taxonomic resolution, phylogeography and phylogeny. For taxonomic resolution and further study on population genetic structure of *C. fieldii*, we sequenced the complete mitogenome of *C. fieldii* and analyzed the phylogenetic relationships of Pieridae based on mitogenome data.

Specimens of *C. fieldii* were collected from Taihe County, Jiangxi Province, China (26°49′N, 114°52′E, August 2019) and were stored in Institute of Plant Protection of Qinghai Academy of Agricultural and Forestry Sciences (please contact Dr. Yunxiang Liu, email: 17791394452@163.com) under the voucher number QHAF-ECF02. Total genomic DNA was extracted from muscle tissues of the thorax using DNeasy DNA Extraction kit (Qiagen, Hilden, Germany). A pair-end sequence library was constructed and sequenced using Illumina HiSeq 2500 platform (Illumina, San Diego, CA), with 150 bp pair-end sequencing method. A total of 26.6 million reads were generated and had been deposited in the NCBI Sequence Read Archive (SRA) with accession number SRR14560146. With the mitochondrial genome of *Colias erate* (KP715146) employed as reference, raw reads were assembled using MITObim v 1.7 (Hahn et al., 2013). By comparison with the homologous sequences of other Pieridae species from GenBank, the mitogenome of *C. fieldii* was annotated using software GENEIOUS R11 (Biomatters Ltd., Auckland, New Zealand).

The complete mitogenome of *C. fieldii* is 15,150 bp in length (GenBank accession no. MT371042), containing the typical set of 13 protein-coding, two rRNA and 22 tRNA genes, and one non-coding AT-rich region. The overall base composition of the mitogenome was estimated to be A 39.8%, T 41.2%, C 11.4% and G 7.6%, with a high A + T content of 81.0%. Except for *cox1* started with CGA, all other PCGs started with the standard ATN codons (seven ATG, four ATT and one ATC). Most of the PCGs terminated with the stop codon TAA or TAG, whereas *cox1*, *cox2*, *nad5* and *nad4* end with the incomplete codon T––. Gene order was conserved and identical to most other previously sequenced Pieridae butterflies (Park et al. 2012; Hao et al. 2013; Cao et al. 2016; Fang et al. 2016; Nie et al. 2018). The 22 tRNA genes vary from 60 bp (*trnS1*) to 70 bp (*trnK*). Two rRNA genes (*rrnL* and *rrnS*) locate at *trnL1*/*trnV* and *trnV*/control region, respectively. The lengths of *rrnL* and *rrnS* in *M. calida* are 1327 and 770 bp, with the AT contents of 85.0% and 84.9%, respectively.

Phylogenetic analysis was performed based on the nucleotide sequences of 13 PCGs from 21 Papilionoidea species. Phylogenetic tree was constructed through raxmlGUI 1.5 (Silvestro and Michalak 2012). Results showed that *C. fieldii* is indeed the sister species of *Colias erate* with a high support value (BS = 100) (Figure 1). All seven Coliadinae species formed one clade and was sister to Pierinae butterflies. Within Coliadinae, the relationships (*Eurema* + (*Gonepteryx* + (*Catopsilia* + *Colias*))) were highly supported, and similar results were found in the previous work (Ding and Zhang 2017; Zhou et al. 2020). In conclusion, the mitogenome of *C. fieldii* is sequenced in this study and can provide essential DNA molecular data for further phylogenetic and evolutionary analysis of Pieridae.

**Figure 1.**
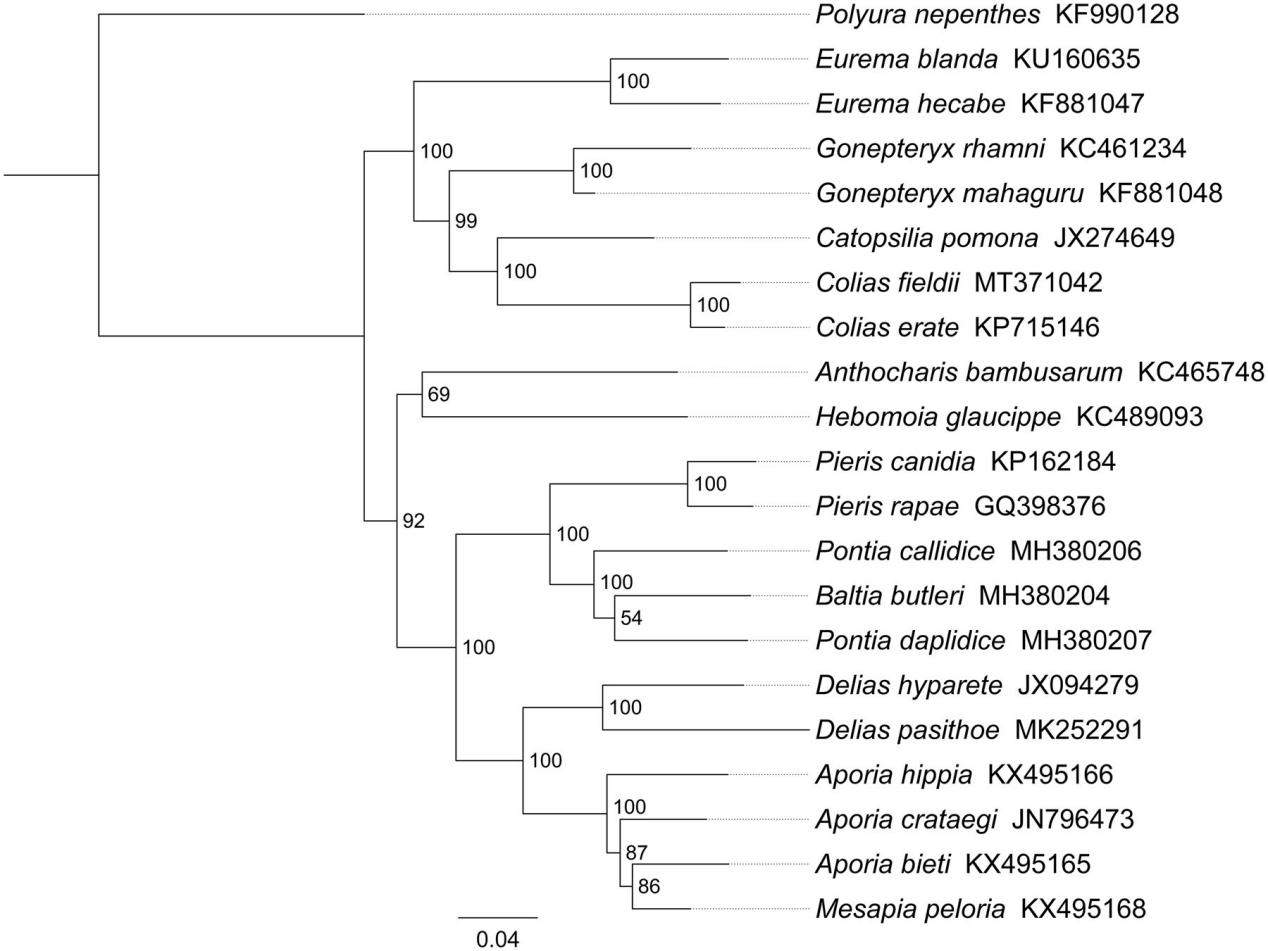
Phylogenetic relationships based on the 13 mitochondrial protein-coding genes sequences inferred from RaxML. Numbers on branches are Bootstrap support values (BS).

## Data Availability

The data that support the findings of this study are openly available in NCBI (National Center for Biotechnology Information) at https://www.ncbi.nlm.nih.gov/, reference number MT371042. The associated BioProject, SRA, and Bio-Sample numbers are PRJNA730322, SRR14560146, and SAMN19228343 respectively.
